# Vacuum-Assisted Osmotic Dehydration of Autumn Olive Berries: Modeling of Mass Transfer Kinetics and Quality Assessment

**DOI:** 10.3390/foods10102286

**Published:** 2021-09-27

**Authors:** Mohamed Ghellam, Oscar Zannou, Charis M. Galanakis, Turki M. S. Aldawoud, Salam A. Ibrahim, Ilkay Koca

**Affiliations:** 1 Food Engineering Department, Faculty of Engineering, Ondokuz Mayis University, 55000 Samsun, Turkey; mohamed.gh2010@gmail.com (M.G.); zannouoscar@gmail.com (O.Z.); itosun@omu.edu.tr (I.K.); 2 Research & Innovation Department, Galanakis Laboratories, 73100 Chania, Greece; 3 Food Waste Recovery Group, ISEKI Food Association, 1190 Vienna, Austria; 4 Department of Botany & Microbiology, College of Science, King Saud University, Riyadh 11451, Saudi Arabia; tdawoud@ksu.edu.sa; 5 Food and Nutritional Sciences Program, North Carolina A&T State University, Greensboro, NC 27411, USA; ibrah001@ncat.edu

**Keywords:** *Elaeagnus umbellata*, osmotic dehydration, modeling, diffusion coefficients, quality characterization

## Abstract

Autumn olive fruits were osmo-dehydrated in sucrose solution at 70 °C under vacuum and atmospheric pressure. The mass transfer kinetics data were applied to the models of Azuara, Crank, Page, and Peleg. The Peleg model was the best-fitted model to predict the water loss and solid gain of both treatments. The vacuum application decreased the effective diffusivities from 2.19 × 10^−10^ to 1.55 × 10^−10^ m^2^·s^−1^ for water loss and from 0.72 × 10^−10^ to 0.62 × 10^−10^ m^2^·s^−1^ for sugar gain. During the osmotic dehydration processes, the water activity decreased and stabilized after 5 h, while the bulk densities increased from 1.04 × 10^3^ to 1.26 × 10^3^ kg/m^3^. Titratable acidity gradually reduced from 1.14 to 0.31% in the atmospheric pressure system and from 1.14 to 0.51% in the vacuum system. pH increased significantly in both systems. Good retention of lycopene was observed even after 10 h of treatments. For the color parameters, the lightness decreased and stabilized after 30 min. In comparison, the redness and yellowness increased in the first 30 min and gradually decreased towards the initial levels in the fresh fruit.

## 1. Introduction

The role of food bioactives to protect consumers’ health has been upgraded after the outbreak of the COVID-19 pandemic [1,2]. Autumn olive fruit (*Elaeagnus umbellata*) are delicious red berries native to Southern Europe and Western and Central Asia and were recently introduced to many countries worldwide. They are a good source of various phytonutrients [3]. The fruits’ lycopene content can reach 54 mg in 100 g fresh fruits, 15 times higher than the lycopene content in tomatoes [4]. Autumn berries are widely thought to protect human health from several diseases and some cancers (e.g., prostate cancer). These sweet-tart berries can be consumed fresh or used for preserves, fruit rolls, juice, and other food products [3,5,6,7].

Due to their high water content, autumn berries and many fruits and vegetables are more subjected to perishability after the harvest [4]. Many technologies are used to preserve these foodstuffs and to increase their shelf-lives [8,9]. Osmotic dehydration is one of the most common technologies used for fruit preservation [10,11]. It can be applied as a primary treatment or as pretreatment of many subsequent treatments, including freezing, hot air-drying, and freeze-drying [12,13]. The osmotic dehydration technique consists of immersing the foodstuffs into hypertonic solutions or highly concentrated solutions (e.g., sugar and salt solutions). During the process, two main mass fluxes co-occurred due to the driving force between media. Water outflow occurs from the food matrix to the surroundings and the solute inflow from the food solution. Moreover, some natural solutes such as sugars, vitamins, and organic acids of the processed product can leach at negligible quantity compared to other fluxes [13].

Osmotic treatment is the desired technique since it improves the texture and stability of the pigments during dehydration and storage [13] and prevents the decline of dried foods’ nutritional value [14]. High-quality dried fruits and vegetables are good products for a large market where fresh-like and healthy products are highly needed. The various parameters, including solution concentration, temperature, treatment time, food-to-solution ratio, food nature, and geometry, play essential roles in the mass transfer mechanism between solution and osmo-dehydrated food [4,15]. To enhance the efficiency of mass transfer, numerous researchers suggested the combination of osmotic process and other novel techniques such as vacuum, microwave, centrifugal force, and high-intensity ultrasound [16,17,18,19]. Furthermore, many investigations have been carried out better to understand the mass transfer kinetics during osmotic dehydration—for example, the modeling of the mass transfer mechanism to predict water loss and solute gain evolutions. The mass diffusion coefficients and the equilibrium values have been calculated based on the mathematical equations [19,20,21].

Physico-chemical properties of the final product are primordial responses to evaluate process effects and the quality of the final food. The leakages of food natural components (acids and sugars), retention and stabilization of sensitive elements (vitamins and antioxidants), and physical and chemical characteristics changes (density, acidity, and pH) are essential factors to be considered [22,23,24]. Generally, the final product’s quality is closely linked not only to the raw material but also to the processing parameters (temperature, solute, duration, etc.) and the combination of the techniques.

To the best of our knowledge, no research has studied the kinetics and characteristics of autumn olive berries during the osmotic dehydration process. Thus, the present study’s main objective was to investigate and model the kinetics of mass transfer at both atmospheric and vacuum pressures. Furthermore, the water and solute effective diffusivities during osmotic treatment were evaluated. Characteristic changes of density, water activity, pH, titratable acidity, color, and lycopene content in osmo-dried autumn berries were investigated.

## 2. Material and Methods

### 2.1. Fruit Preparation and Osmotic Dehydration Treatments

Autumn olive fruits (*Elaeagnus umbellata*) were harvested from the Agriculture Department’s trial trees, Ondokuz Mayis University, Samsun, Turkey. The fruits were sorted, and those with similar maturity (ripe red fruits) were collected and filled in refrigerator bags (ca. 300 g). Afterward, they were immediately frozen at −20 °C. Before the analyses and osmotic dehydration process, the frozen fruits were sorted again to remove the damaged or crushed ones. They were left to thaw at room temperature before being gently blotted with paper to remove the superficial humidity.

**Figure 1:**
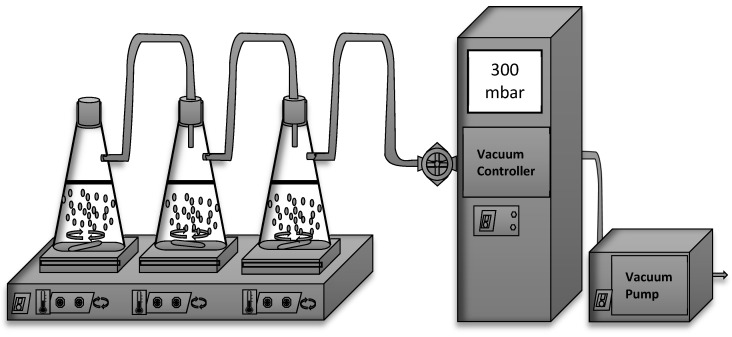
Schematic illustration of the osmotic dehydration process.

Osmotic dehydration was carried at atmospheric pressure and by applying the vacuum. A ratio of 1.8:10 of fruit to the solution (*w*/*w*) was put in 500 mL beakers containing 400 g of sugar solution of 70% (*w*/*w*, commercial sugar, distilled water). The beakers were placed on a hotplate stirrer (M TOPS, Multi-position, Korea) to operate at 70 °C and connected to a modified vacuum rotary evaporator (Büchi, V-800 controller, V-500 pump, Flawil, Switzerland) (Figure 1). Magnetic stirring was performed at 250 rpm during the process, while the experiment was conducted in different cycles of 0.5, 1, 1.5, 2, 3, 4, 5, and 10 h. The first batch was treated at atmospheric pressure without the application of vacuum. The second batch was subjected to 300 mbar for 5 min vacuum and released at atmospheric pressure after the vacuum was applied again for 10 min. The system was kept closed to create a continuous vacuum until the end of the process. After the osmotic treatments, the samples were gently taken from the osmotic solutions and rinsed with distilled water (ca. 20 s). They were immediately blotted with paper to remove the excess surface water (2–3 min). Each beaker presented a repetition, and each repetition had two or more replications for the analyses.

### 2.2. Analyses

Water activity (a_w_) was determined using a calibrated water activity meter at 25 °C ± 0.1 (Aqualab, 4TE, Pullman, WA, USA). To resolve moisture and dry matter, a quantity of fruits (ca. 5 g) was dried at 70 °C in a laboratory drying oven (NÜVE, FN 500P, Turkey) until constant weight. Total soluble solids were analyzed by an Abbe refractometer (Model DTM-1, Atago, Tokyo, Japan). The CIE L*a*b* scale of a digital colorimeter (Model CR-400, Minolta-Konica Sensing Inc., Osaka, Japan) was used to measure fruit color. Five readings were taken for each osmotic treatment repetition (3 repetitions). The total color change (ΔE) after the application of the treatments was determined with Equation (1):

(1) $$\Delta E={\left({\left(\Delta L^{*}\right)}^{2}+{\left(\Delta a^{*}\right)}^{2}+{\left(\Delta b^{*}\right)}^{2}\right)}^{\frac{1}{2}}$$

where ΔL*, Δa*, and Δb* are the difference of L*, a*, and b* before and after drying, respectively.

The fruit diameter was taken as the mean of length and width measured with a digital caliper (TRESNA, Series: EC16, Guangxi Province, China). Lycopene content was spectrophotometrically determined using the previous procedure of Ghellam et al. [4], and the mean was the value of 6 repetitions. pH was measured in duplication for each repetition and triplicate for the fresh fruits using a pH meter (Model Starter 3100, OHAUS, Parsippany, NJ, USA). The titratable acidity was determined based on the potentiometric method. Briefly, 10 g of berries was combined with 100 mL of distilled water and extracted for 12 h in the refrigerator. The mixture was filtered, a volume of 10 mL was taken and titrated with 0.1 M NaOH to pH 8.1 and the concentration of acid was expressed as % of malic acid. The bulk density (ρ_b_) of fruits was measured according to Yang and Atallah [25]. The weight and volume of fruits were measured using analytical balance (RADWAG, AS 220/C/2, Radom, Poland) and measuring a cylinder filled with distilled water, and the weight per volume was expressed as bulk density (kg/m^3^). Measurements of the fruits’ color were calculated after 15 repetitions; length and width after 20 repetitions; density, water activity, titratable acidity, and pH after 3 repetitions; total soluble solids after 5 repetitions; and moisture content after 8 repetitions. The initial fresh fruits’ characteristics are shown in Table 1.

### 2.3. Mass Transfer Parameters

The mass transfer of fruit was determined according to the previous method [26] as a function of water loss (WL), sugar gain (SG), and weight reduction (WR). The water loss was defined as the net loss of water from autumn olive fruits (Equation (2)), the sugar gain as the net gain in total solids based on the initial mass (Equation (3)), and the weight reduction as the net mass reduction of fruits on the initial mass basis (Equation (4)).

(2) $$\mathrm{WL}=\frac{W_{i}X_{i}-W_{f}X_{f}}{W_{i}}\times 100$$

(3) $$\mathrm{SG}=\frac{W_{f}(1-X_{f})-W_{i}(1-X_{i\;})}{W_{i}}\times 100$$

(4) $$\mathrm{WR}=\frac{W_{i}-W_{f}}{W_{i}}\times 100$$

where WL is the water loss (g water/g initial mass of autumn fruits)%, SG is the sugar gain (g sugar/g initial mass of autumn fruits)%, WR is the mass reduction (g/g of the initial mass of autumn fruits)%, *Wi* is the initial mass of autumn fruits (g), *Wf* is the mass of autumn fruits after osmotic dehydration or at a specific time (g), *Xi* is the water content as a fraction of the initial mass of autumn fruits, and *Xf* is the water content as a fraction of the mass of fruits after osmotic dehydration.

### 2.4. Empirical Models for Osmotic Dehydration

Mass transfer kinetics during osmotic dehydration of autumn olive fruits at both atmospheric pressure and vacuum were modeled according to Peleg, Azuara, Page, and Crank equations. The validation of the models was checked by a nonlinear regression method (Excel Solver, Microsoft 2016, Redmond, WA, USA), considering the kinetics of water loss (WL) and solids gain (SG) in the function of dehydration time. The coefficient of determination (R^2^) and root mean square error (RMSE) were calculated to show the adequacy of models and the level of fitting between experimental and predicted values.

#### 2.4.1. Peleg’s Model

Peleg’s model was used to describe the dehydration curves, which asymptotically approaches equilibrium [27].

(5) $$\mathrm{WL}\left(t\right)={\mathrm{ML}}_{0}\pm \frac{t}{k_{1w}+k_{2w}t}$$

(6) $$\mathrm{SG}\left(t\right)={\mathrm{SG}}_{0}\pm \frac{t}{k_{1s}+k_{2s}t}$$

where WL(t) and SG(t) are loss of water (%) and solute gain (%) at t time(s), respectively, and k_1*w*_, k_2*w*_, k_1*s*_, k_2*s*_ are kinetic constants. Wl_0_ and SG_0_ are equal to 0 at t = 0. Moreover, 1/k_2*m*_ and 1/k_2*s*_ represent the value of water loss and solid gain at equilibrium (t = ∞).

#### 2.4.2. Azuara’s Model

The model proposed by Azuara et al. [12] was used to model water loss and solid gain. This model is based on the mass balance during osmotic dehydration to estimate the dehydration equilibrium point’s mass transfer parameters. The equations of a model for water loss and solid gain were given as follows:

(7) $$\mathrm{WL}\left(t\right)=\frac{\;S_{w}\;t\;{\left({\;\mathrm{WL}}_{\infty \;}\right)}_{\;}}{1+S_{w}\;t\;}$$

(8) $$\mathrm{SG}\left(t\right)=\frac{S_{s\;\;}t\;\left({\;\mathrm{SG}}_{\infty }\right)}{1+S_{s}\;t}$$

where WL(t) and SG(t) are loss of water (%) and solute gain (%) at t time(s), respectively. S*_w_* and S*_s_* constants are related to the water and solute diffusion out and into the fruit. WL_∞_ and SG_∞_ are the considering quantities at equilibrium (t = ∞). The equilibrium points (WL_∞_ and SG_∞_) and diffusion constants (S*_w_* and S*_s_*) are respectively calculated from the reciprocal slopes and intercepts of (t/WL(t)) and (t/SG(t)) versus t (s).

#### 2.4.3. Crank Model and Effective Diffusivity

Fick’s second law was also used to model the dehydration kinetics. The crank model is the solution for Fick’s equation [28] for constant process conditions. It is considered that the autumn olive berry is a complete sphere (r = 3.29 mm) with initially uniform water and solute contents, as proposed by Azoubel and Murr [20] for sphere geometry fruit.

(9) $$\mathrm{Wr}\;\mathrm{or}\;\mathrm{Sr}=\frac{6}{\pi^{2}}\overset{\infty }{\underset{n=1}{\sum }}\frac{1}{\;n^{2}}\;exp\left(-n^{2}\pi^{2}D_{e}\;\frac{t}{r^{2}}\;\right)$$

(10) $$\mathrm{Wr}=\frac{\mathrm{WL}\left(t\right)-{\mathrm{WL}}_{\infty \;}}{{\mathrm{WL}}_{0\;}-{\;\mathrm{WL}}_{\infty \;}}$$

(11) $$\mathrm{Sr}=\frac{\mathrm{SG}\left(t\right)-{\;}_{\;}{\mathrm{SG}}_{\infty \;}}{{\;\mathrm{SG}}_{0\;}-{\;\mathrm{SG}}_{\infty \;}}$$

where Wr and Sr are the dimensionless amounts of water loss and solid gain, and WL_∞_ and SG_∞_ are the equilibrium amounts (calculated from Peleg’s equation) for water loss and solid gain, respectively. Wl_0_ and SG_0_ are equal to 0 at t = 0. D_e_ is the effective diffusivity (m^2^·s^−1^), n is the number of series (30 terms are used for the calculations), *r* is the sphere radius, and *t* is the time (s).

#### 2.4.4. Page’s Model

Page’s model is one of the most used empirical models to predict dehydration processes’ mass transfer. It is an empirical modification of a simple exponential model, given by the following equation:

(12) $$\mathrm{Wr}\;\mathrm{or}\;\mathrm{Sr}=exp\left(-A\;\;.\;t^{B}\right)$$

where *A* and *B* are model constants, and *t* is the time (s).

### 2.5. Statistical Analysis

Statistical significance (*p* < 0.05) was determined by the analysis of variance (ANOVA) using SPSS statistics software (version 23). Paired-sample T-tests determined the statistical difference between the means of the two treatments (atmospheric and vacuum pressures) at a specific time (t) after splitting into corresponding time groups.

## 3. Results and Discussion

First, some physicochemical properties of fresh autumn olive berries were shown in Table 1. The obtained results were very close to our previous studies and others in the literature [3,5,6,7,29,30]. These small red berries are a rich source of lycopene and have a high-water content. The osmotic dehydration process is one of the successful ways to reduce the perishability of fruits and preserve their functional compounds. In the present work, the optimal conditions previously found [4] are applied to study the kinetics of osmotic dehydration of autumn olive berries under low and atmospheric pressures.

**Figure 2:**
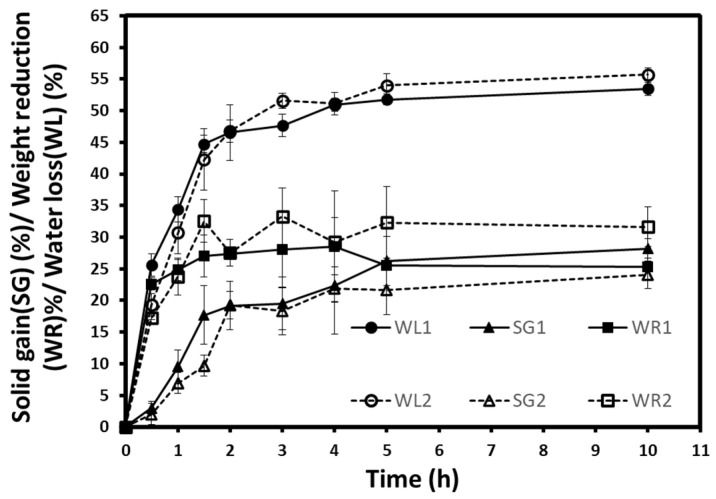
Water loss, solid gain, and weight reduction of autumn berries during osmotic dehydration at atmospheric pressure (1) and vacuum pressure (2). Each point represents the mean and standard deviation of a duplicate of three repetitions.

The results of the kinetics of water loss (WL), solid gain (SG), and weight reduction (WR) obtained during the osmotic dehydration of autumn berries at atmospheric pressure (1) and vacuum (2) are shown in Figure 2. As can be seen, all mass transfer parameters presented an increase during both osmotic treatments. The increases in WL, WR, and SG are desirable in the osmotic treatments of fruits, vegetables, or other foodstuffs. However, these increases can have different rates depending on treatment conditions and the food’s intrinsic characteristics [18,19,31]. The statistical analysis of the three parameters (WL, SG, WR) were performed. There was a significant difference between the two processes in WL at 0.5, 1, and 3 h; in WR at 0.5 and 3 h; and in SG at 3 h. Despite that, WL1 and WL2 seem close, and SG2 is lower than SG1. This can be confirmed by the weight reduction (WR) curves where WR2 values showed a high rate, especially during the last hours. Moreover, the WL/SG ratio is another criterium to consider since it reflects the mass transfer amount, the amount of outflow water, and the number of inflow sugars. The higher ratio means a higher WL or a lesser SG. WL2/SG2 presented higher values than WL1/SG1, which clearly explained the lesser SG during vacuum osmotic treatment. According to these findings, vacuum dehydration may have similar effects on water loss, but it appears to exhibit an opposite effect by uptaking the soluble solids.

### 3.1. Evaluation of Models and Effective Diffusivities

**Figure 3:**
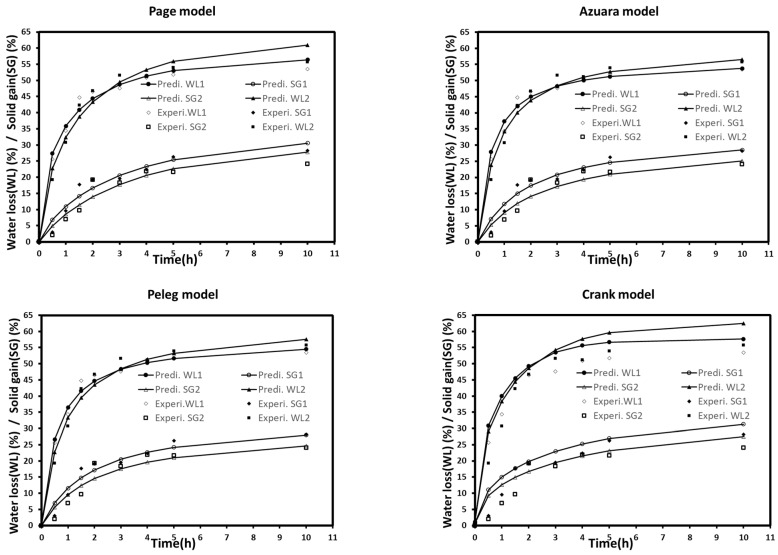
Experimental data and predicted models’ lines (Peleg, Azuara, Page, and Crank model) of water loss (WL) and sugar gain (SG) at atmospheric (1) and vacuum (2) pressures. Each point represents the mean of a duplicate of three repetitions.

The kinetics of mass were analyzed using kinetic models to obtain a better insight into the osmotic dehydration of autumn berries with and without the application of a vacuum. Models and fitting parameters are given in Table 2. The fitted curves for all models are shown in Figure 3. As shown in Table 2, Peleg, Azuara, Page, and Crank models were the better predictors for water loss for both treatments (WL1 and WL2), where R^2^ and RMSE values were more than 0.974 and less than 2.56%, respectively. This indicated that these models were highly adequate and reliable to predict osmotic dehydration for water loss. In general, SG gave lower R^2^ values when compared to WL. The lowest R^2^ values were found in SG2, where the Crank model gave an R^2^ value of 0.885. This can be clearly seen in Figure 3, which shows several deviations between the experimental data of SG2 and model-predicted lines. The Peleg model was found to be the best-fitted model for SG1 and SG2.

The predicted water loss at equilibrium point (WL_∞_) described by the Peleg and Azuara equations (62.68% and 60.92%) showed an increase by the application of vacuum (Table 2). However, the highest solid gains (SG_∞_) were obtained at atmospheric pressure treatment (33.25%, 33.86%) with Peleg and Azuara models. Similarly, Deng and Zhao [17] reported higher water loss values with the application of a pulsed vacuum compared to a single-agitation osmotic dehydration. Furthermore, a comparison of vacuum treatment with atmospheric pressure impregnation had revealed that the application of vacuum had induced the solute gain and water loss in papaya fruit [32,33]. During vacuum osmotic treatment, the hydrodynamic mechanism permits to increase the internal gas/external solution exchange [18,32]. In spite of the driving force between both media (70° Brix solution), these contradicted results suggest that a higher water loss might lead to a reduction of the available water to dissolve solute and consequently to low sugar penetration. The gas expansion plays a crucial role in vacuum osmotic dehydration as it increases liquid penetration into emptied food pores after the release at atmospheric pressure [18]. Thus, the fruit tissue difference and the unreleased atmospheric pressure (continuous vacuum) could be amongst the factors that caused these results.

As shown in Table 2, with the vacuum application, an increase of Peleg k_1_ constant was observed for both WL and SG. However, an opposite trend can be observed for the Azura S constant and Page A constant. These decreases reflect a possible effect of vacuum to decrease the overall water and solute diffusion rate. This can be confirmed by the effective diffusivities calculated from the Crank equation. The effective diffusivity of water (Dew) and solute (Des) decreased from 2.19 × 10^−^^10^ m^2^·s^−1^ to 1.55 × 10^−10^ m^2^·s^−1^ and from 0.72 × 10^−10^ m^2^·s^−1^ to 0.62 × 10^−10^ m^2^·s^−1^, respectively, by the application of low pressure. Previously, Zielinska et al. [19] found that D_ew_ ranged from 0.7× 10^−10^ m^2^·s^−1^ to 6.1 × 10^−10^ m^2^·s^−1^ and D_es_ ranged from 0.4 × 10^−10^ m^2^·s^−1^ to 3.7× 10^−10^ m^2^·s^−1^ for cranberries subjected to osmotic dehydration in sucrose solution. Furthermore, Panades et al. [30] reported an analogous interval of effective diffusivities ranging from 0.69 × 10^−10^ m^2^·s^−1^ to 1.47× 10^−10^ m^2^·s^−1^ for osmo-dehydrated guava fruits in sucrose solution (65° Brix). In contrast, Fito [18] reported that the application of low pressures increased mass transfer rates. This difference can probably be engendered firstly due to the characteristics of treated fruits (type, shape, whole fruits). Secondly, the continuous vacuum osmotic dehydration can reduce the dehydration rate and solids penetration. The possible resistance resulted in the creation of a vacuum inside food tissues or the favored compaction of tissues [17].

### 3.2. Kinetics

#### 3.2.1. Water Activity

**Figure 4:**
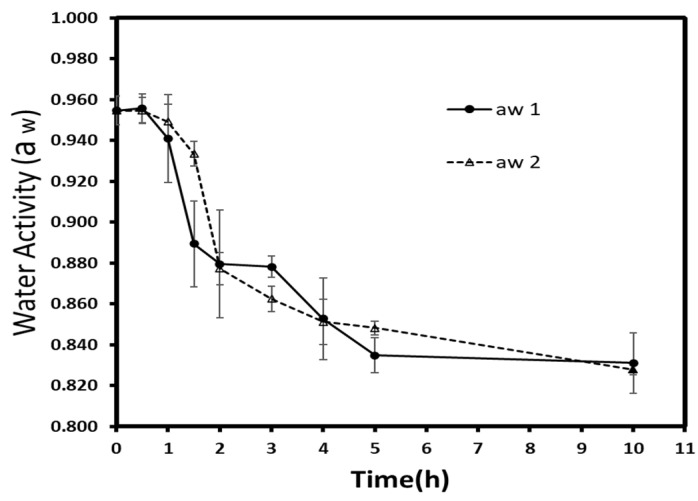
Water activity evolution during osmotic dehydration at atmospheric (a_w_ 1) and vacuum (a_w_ 2) pressures. Each point represents the mean and standard deviation of duplicate of three repetitions.

Osmotic dehydration is a pathway to reduce the water activity (a_w_) of fruits and vegetables. By lowering the available water content or promoting its interaction with solutes (salts, sugars), this process reduces the microbial growth and increases the shelf-life of treated material [34]. Thus, a_w_ is considered as an essential parameter to understand perishability more and to control food storage. Autumn berries had an initial a_w_ of 0.955 (±0.007), and the water activity kinetics are given in Figure 4. Generally, each treatment presented a significant decrease (*p* < 0.05) during osmotic dehydration. Concerning the difference within time groups, a significant reduction (*p* < 0.05) was observed for atmospheric pressure over vacuum-treated samples at 1.5 h. Afterward, no significant difference was detected, but at 10 h, the atmospheric pressure (0.831 ± 0.015) and vacuum- treated samples (0.828 ± 0.002) demonstrated again a statistically significant difference (*p* < 0.05).

The water activity level was expected to be lower at low pressures since the WL/SG ratio of vacuum treated samples had shown a higher value. Not far from this finding, Moreno et al. [33] reported the non-significant effect of low pressure on the a_w_ of dehydrated papaya in sucrose solution. The low sugar penetration should be another factor since the interaction between sugar penetration and water is known to reduce the a_w_.

#### 3.2.2. Density

**Figure 5:**
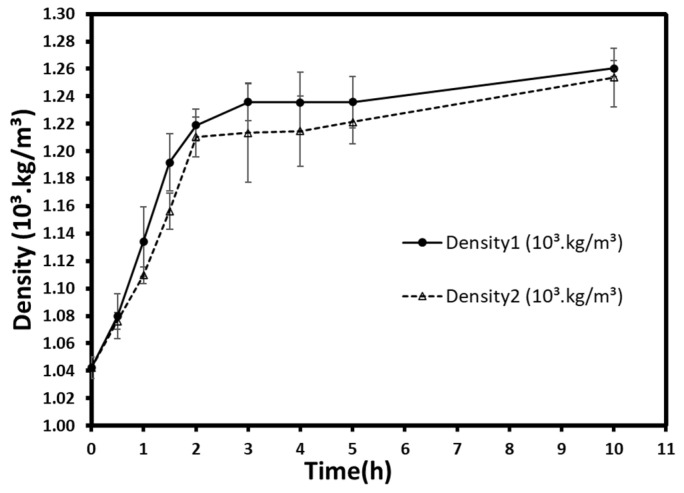
Density evolution during osmotic dehydration at atmospheric (density 1) and vacuum (density 2) pressures. Each point represents the mean and standard deviation of a duplicate of three repetitions.

The densities varied from 1.04 × 10^3^ kg/m^3^ at t = 0 h (fresh berries) to 1.26 × 10^3^ kg/m^3^ and 1.25 × 10^3^ kg/m^3^ after 10 h for atmospheric and low-pressure treatments, respectively (Figure 5). A rapid and remarkable increase in density was observed during the first 3 h and began to slow down gradually at the end of the treatments. In general, the density at atmospheric pressure seemed to be higher, but no significant differences (*p* > 0.05) were detected between the two pressures for all time groups. Despite the low sugar uptake previously mentioned, which may reduce density’s increase, the vacuum-osmosed samples appear to have a density similar to that of normal pressure. This could result in a slight difference, which has no significant effect on density. Otherwise, the gas flow during the vacuum application could compensate for this uptake and obtain similar density values.

Generally, the osmotic treatment increases the bulk density of treated fruits and vegetable. Many studies have proven that, if not shrinkage, the mass uptake during osmosis increases fruits’ weight per volume. For instance, the pre-treatment of fresh persimmon with ultrasound and osmotic dehydration and the subsequent convective drying showed an increase in weight, and consequently in density, which reached up to 1.020 g/mL [35]. Moreover, the impregnation of apple slabs in sucrose solutions increased the density from ≈0.82.10^3^ kg/m^3^ to over 0.90 ×10^3^ kg/m^3^ [36]. Likewise, osmoconcentration of blueberries increased their densities from 0.493 ×10^3^ kg/m^3^ to 0.709 ×10^3^ kg/m^3^ [37].

#### 3.2.3. Titratable Acidity and pH

**Figure 6:**
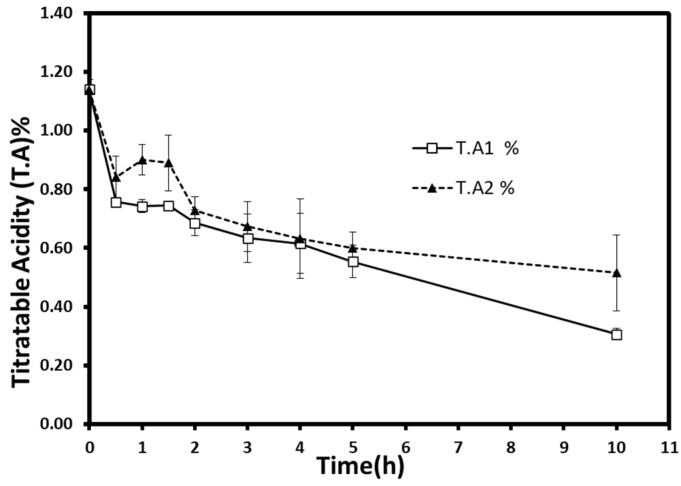
Titratable acidity (malic acid%) evolution during osmotic dehydration at atmospheric (T.A1) and vacuum (T.A2) pressures. Each point represents the mean and standard deviation of a duplicate of three repetitions.

Weight reduction during the process was taken into account to calculate the titratable acidity or the dominant malic acid content. Titratable acidity decreased from 1.14% (malic acid%) in fresh berries to 0.31% (±0.02) and 0.51% (±0.13) for atmospheric pressure and vacuum samples, respectively (Figure 6). Interestingly, about 40% of the acid decrease occurred during the first two hours. It started to slow down slightly to reach more than 54% of acid loss at the end of the process. Statistical analysis showed a significant acid decrease (*p* < 0.05), but no significant difference was found within time groups.

Malic acid, alongside many organic acids (e.g., citric acid, ascorbic acid), is affected during the osmotic process due to their water solubility and leaching of water. Moreover, these acids’ chemical degradation and leakage can be intensified by high-temperature procedures [23]. Thus, the loss of organic acids is a phenomenon that is highly expected to appear for all osmotic treatments. Furthermore, the leakage of these substances can be accelerated by the hydrodynamic mechanism using ultrasonic power or pulsed-vacuum technique [17,38]. However, no comparable results were obtained in this work. The pressure reduction (300 mbar) did not show any effect on acid loss. This could be related to the similar water losses obtained at both pressures. Additionally, the continuity and/or insufficiency of applied vacuum during the dehydration process might be the reason behind the insignificant effect.

The simultaneous solute uptake and acid loss could increase the sugar-to-acid ratio. The increase of the sugar-to-acid ratio in osmovac-dried apple slices could bring a more pleasing taste and retain the fresh fruit flavors [39]. Thus, these kinds of products can be used as high-quality ingredients in snacks, breakfast, and rehydrated foods. However, a high sugar uptake and the candying effect could harm the nutritional profile and the acceptability of long-period treated products [40]. Several methods can avoid a drastic decrease in acids. For example, by adding industrial acids, many organic acids (e.g., citric acid) can be used in a dehydrating medium to maintain the acidity if desired. The reuse of the osmotic solution for many process cycles is also suggested to partially reduce acid losses [31].

**Figure 7:**
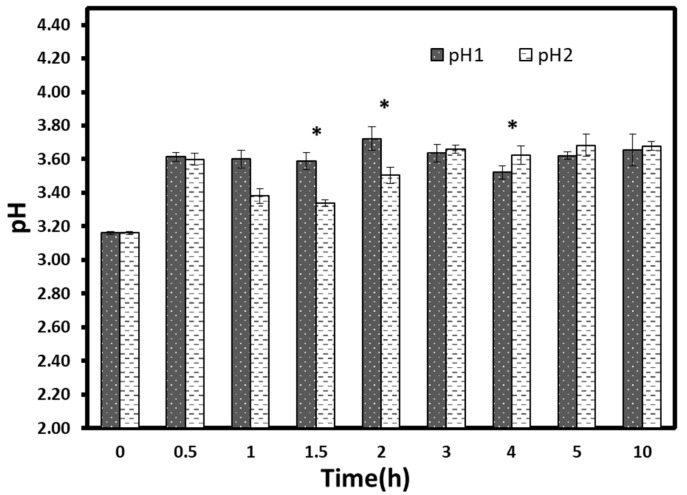
pH evolution during osmotic dehydration at atmospheric (pH 1) and vacuum (pH 2) pressures. Each point represents the mean and standard deviation of a duplicate of three repetitions. * Significant difference between time groups.

The results of pH variations during osmotic processes were shown in Figure 7. The pH value of the non-processed fruit was 3.16 ± 0.01. A significant increase (*p* < 0.05) was detected for both treatments in all hours (≈ 3.6). However, only two significant low pH values were obtained at t = 1.5 h and t = 2 h for vacuum treated samples compared to typical pressure samples. Generally, starting from 2 h, pH values seemed to have a kind of stabilization, irrespective of treatment time. The increase of pH in the first hours might be ascribed to the abrupt reduction of acid content. The pH stabilization became early before the titratable acidity stabilization occurred. This result can be explained by the fact that the organic acids are weak acids; thus, the titratable acidity variation does not always reflect the pH variation [24]. A similar result was obtained in a previous work by Moreno et al. [33]. They reported that the application of vacuum during osmotic dehydration had favored the mass transfer without significant pH changes.

#### 3.2.4. Lycopene Retention

Lycopene is a well-known antioxidant and natural pigment [3,41,42]. The lycopene content after 10 h of osmotic proceeding is indicated in Table 3. The osmo-dehydrated berries presented significant, high contents of lycopene (>24 mg/100 g F.W) compared to the fresh berries. However, while considering the weight reduction, lycopene did not significantly differ from the fresh fruit. This result can be explained by a concentration of lycopene after a loss of water during osmotic processes. Furthermore, despite the type of treatment and the long duration (10 h), the lycopene content did not change. In the present study, lycopene content in fresh berries was within a range previously shown in studies [3,4,5]. Moreover, our results agreed with Tonon et al. [14] and Heredia et al. [22] for the osmotically dehydrated tomato and cherry tomato, respectively.

#### 3.2.5. Color

The variation of color parameters (L*, a*, b*, and ∆E) during 10 h of the osmotic process for both atmospheric pressure and vacuum are shown in Table 4. There was a significant decrease (*p* < 0.05) of lightness (L*) of fresh berries (31.53 ± 1.22) during the osmotic dehydration process (*p* < 0.05). The dehydrated samples under vacuum showed stabilization regardless of the dehydration time, showing significant low values of L* compared to atmospheric pressure. The processing at atmospheric pressure induced a more substantial decrease in long-term treatments (t = 5 h, t = 10 h). In contrast, redness (a*) and yellowness (b*) showed significant increases, especially at the beginning of the two processes (t = 0.5 h). These values did not show any stabilization and began to decrease slightly as the treatment progressed. At t = 10 h, the values were very close to fresh fruit for a* or somewhat higher for b*. This tendency was manifested by increasing variation in the early hours and reducing color change (∆E) in the final hours. The primary significant differences between osmotic dehydration at atmospheric pressure and under vacuum were encountered at t = 0.5 h, t = 1.5 h, and at t = 10 h.

The color variation could not be attributed to the leakage of lycopene of autumn olives regarding the findings on the lycopene content. However, the concentration of lycopene may lead to changes in the fruit’s opacity and redness levels. These results agree with the findings of Heredia et al. [22], who have mentioned the lightness reduction. These authors reported that the increase of opacity in osmosed cherry tomato could be attributed to the shrinkage resulting from water loss and sugar gain. It was observed that the redness and yellowness increased at the beginning of the experiment and became more or less stable after 6 h depending on the applied treatments.

Moreover, Falade et al. [21] have studied the osmo-dehydration behavior of watermelon slices and reported increasing color parameters (L*, a*, b*). They affirmed that red pigment concentration was related to the water removal for both osmosed and osmo-oven-dried samples. The vacuum application affected the increase in opacity since the vacuum impregnation of the papaya had led to low L* values due to the loss of air and its substitution by the osmotic solution [33]. The osmosed autumn berries still have an excellent appearance of color and shape.

## 4. Conclusions

Mass transfer parameters (water loss and sugar gain) of autumn berries were studied during osmotic dehydration at atmospheric pressure and under vacuum. Kinetic modeling showed that the Peleg model was the best-fitted model. The Peleg and Azuara models’ equilibrium points had higher predicted water loss and lower sugar gain values in a vacuum than atmospheric pressure. The diffusion coefficients showed a similar downward trend under vacuum osmotic dehydration. Effective diffusivities calculated by the Crank model confirmed this by a decrease from 2.19 × 10^−10^ m^2^·s^−1^ to 1.55 × 10^−10^ m^2^·s^−1^ and from 0.72 × 10^−10^ m^2^·s^−1^ to 0.62 × 10^−10^ m^2^·s^−1^ for water loss and sugar gain, respectively. Water activities began to stabilize after 5 h of treatment, and similar values were obtained at the end of the processes (a_w_ ≈ 0.830). Bulk densities increased to reach 1.26 × 10^3^ kg/m^3^ of density (t = 10). Titratable acidity showed a gradual decrease over time, from 1.14% to 0.31 (±0.02) and 0.51 (±0.13) for atmospheric and low pressures, respectively. pH values increased significantly in both treatments but were still low to reduce microbial contamination. Lycopene was well retained even after 10 h of osmotic dehydration. Analyses of the color showed a decrease in lightness at the beginning of the processes and after stabilization. However, redness and yellowness were highly increased initially and started to decrease gradually to exhibit similar levels to fresh berries at the end of the process. A total of 5 h of osmotic dehydration at atmospheric pressure would be sufficient and optimal to ensure dehydration and to save energy since the values were very close to the equilibrium point.

Briefly, it can be concluded that osmotic dehydration was a useful technique for reducing water content and increasing sugar uptake in autumn olive berries. Therefore, it helps to increase the preservation and shelf life of the final product. In order to ensure more stability, a second dehydration process (sun drying, convective drying, freeze drying, etc.) can be applied to continue reducing the water content and water activity to the required levels of preservation. Vacuum application appeared to decrease the coefficients of diffusion. This can be attributed to the continuous and/or the insufficiency of vacuum-osmotic dehydration, which was not only a functional technique to retain nutrients but also to concentrate them. Moreover, the characteristics of the final products were generally improved. Consequently, high-quality osmosed berries were obtained and are ready to be introduced to the market. Further studies are needed to better understand the effect of low pressure on the mass transfer of these small berries. Furthermore, the combination with other techniques (ultrasound, microwave, etc.) may improve the mass transfer and increase the quality of dehydrated fruits.

## Figures and Tables

**Table 1:** Initial characteristics of non-processed autumn olive fruits.

Characteritics	Mean	S. D
Color parameters:		
L*	31.53 ^a^	1.20
a*	6.10 ^a^	1.23
b*	4.43 ^a^	0.44
Length (mm)	6.65 ^b^	0.51
Width (mm)	6.51 ^b^	0.50
Density 10^3^. kg/m^3^	1.04 ^c^	0.01
Total Soluble Solids (° Brix)	14.80 ^d^	0.27
Water activity	0.955 ^c^	0.007
Moisture content (%, Fresh Weight)	77.24 ^e^	0.41
Titratable acidity (%, Malic acid)	1.14 ^c^	0.03
pH	3.16 ^c^	0.01

Superscripts, ^a^, ^b^, ^c^, ^d^, and ^e^ represent the means (± S, D) of 15, 20, 3, 5, and 8 measurements, respectively.

**Table 2:** Values of the models’ parameters and diffusion coefficients for water loss and solid gain.

	Peleg’s Model					Azuara’s Model				Page’s Model				Crank’s Model		
	k_1_	k_2_	R^2^	RMSE	WL_∞_/SG_∞_	S × 10^−4^	R^2^	RMSE	WL_∞_/SG_∞_	A × 10^−3^	B	R^2^	RMSE	De × 10^−3^	R^2^	RMSE
WL1	36.2	0.017	0.996	1.44	57.67	5.4	0.990	1.65	56.53	7.62	0.592	0.986	0.03	2.19	0.990	0.07
WL2	50.7	0.016	0.992	2.25	62.68	3.6	0.982	2.56	60.92	2.48	0.694	0.974	0.05	1.55	0.975	0.09
SG1	202.4	0.030	0.979	1.94	33.25	1.5	0.958	2.02	33.86	5.80	0.798	0.945	0.07	0.72	0.929	0.11
SG2	254.3	0.033	0.963	2.39	29.91	1.2	0.926	2.49	31.07	2.20	0.893	0.915	0.09	0.62	0.885	0.13

R^2^: determination coefficient (%), RMSE: root mean square error (%), k_1_, k_2_, A, B, S (s^−1^), and De (m^2^·s^−1^): models’ coefficients. WL_∞_ (%) and SG_∞_ (%) are the models’ predicted equilibrium points for water loss (WL) and sugar gain (SG), respectively.

**Table 3:** Lycopene retention after 10 h of osmotic treatment of autumn olive fruits.

	Lycopene mg/100 g F.W (±SD) at t = 10 h	
Sample	Weight Reduction not Considered	Weight Reduction Considered
Fresh fruits	18.96 ± 1.22 ^b^	18.96 ± 1.22 ^a^
Atm. pressure-dehydrated fruits	24.31 ± 2.44 ^a^	19.30 ± 2.50 ^a^
Vacuum-dehydrated fruits	25.81 ± 2.97 ^a^	17.72 ± 1.93 ^a^

Means (±standard deviation) in one column with different letters are significantly different (*p* < 0.05).

**Table 4:** Evolution of color parameters (L*, a*, and b*) during osmotic dehydration for normal and low-pressure treatment.

	Atmospheric Pressure-Dehydrated Berries				Vacuum-Dehydrated Berries			
Time (h)	L *	a *	b *	∆E	L *	a *	b *	∆E
0	31.53 ± 1.23 ^a^	6.10 ± 1.27 ^d^	4.43 ± 0.46 ^f^		31.53 ± 1.23 ^a^	6.10 ± 1.27 ^de^	4.43 ± 0.46 ^f^	
0.5	27.78 ± 1.09 ^bc^	9.65 ± 0.90 ^a^	7.71 ± 0.67 ^a^	6.28 ± 0.62	27.08 ± 0.93 ^b^	10.56 ± 1.25 ^a^	8.61 ± 0.77 ^a^	7.66 ± 0.97
1	28.25 ± 0.74 ^b^	8.01 ± 1.09 ^b^	6.95 ± 0.76 ^b^	4.70 ± 0.98	28.17 ± 1.07 ^b^	8.03 ± 1.09 ^b^	7.33 ± 0.63 ^b^	4.98 ± 1.09
1.5	28.16 ± 0.84 ^bc^	7.49 ± 1.28 ^bc^	6.65 ± 0.74 ^bc^	4.51 ± 0.82	27.10 ± 1.05 ^b^	8.40 ± 0.56 ^b^	7.34 ± 0.45 ^b^	5.86 ± 0.75
2	28.01 ± 0.62 ^bc^	6.70 ± 0.71 ^cd^	5.91 ± 0.40 ^de^	3.95 ± 0.61	27.15 ± 1.16 ^b^	7.42 ± 1.36 ^bc^	6.54 ± 0.85 ^c^	5.30 ± 1.06
3	28.66 ± 1.07 ^b^	7.54 ± 0.85 ^bc^	6.45 ± 0.84 ^bcd^	3.94 ± 1.17	27.36 ± 0.7 ^b^	6.55 ± 0.74 ^cd^	6.13 ± 0.47 ^cd^	4.62 ± 0.60
4	28.44 ± 1.18 ^b^	7.01 ± 1.09 ^cd^	5.95 ± 0.54 ^cde^	3.85 ± 0.76	27.10 ± 0.75 ^b^	6.52 ± 0.87 ^cd^	6.01 ± 0.62 ^cde^	4.84 ± 0.74
5	27.12 ± 1.01 ^cd^	5.89 ± 1.05 ^d^	5.45 ± 0.64 ^e^	4.69 ± 1.00	27.26 ± 1.01 ^b^	5.86 ± 0.82 ^de^	5.68 ± 0.43 ^de^	4.56 ± 0.96
10	26.52 ± 0.68 ^d^	6.70 ± 0.45 ^d^	5.81 ± 0.31 ^de^	5.27 ± 0.70	27.27 ± 0.77 ^b^	5.33 ± 0.90 ^e^	5.47 ± 0.32 ^e^	4.54 ± 0.68

L*, a*, and b* are the color parameters representing the lightness, redness, and yellowness, respectively. Means in one column with different letters (^a^, ^b^, ^c^, ^d^, ^e^) are significantly different (*p* < 0.05). 5 readings were taken for each treatment repetition (3 repetitions).

## Data Availability

Data sharing not applicable.
